# Initial nutritional management during noninvasive ventilation and outcomes: a retrospective cohort study

**DOI:** 10.1186/s13054-017-1867-y

**Published:** 2017-11-29

**Authors:** Nicolas Terzi, Michael Darmon, Jean Reignier, Stéphane Ruckly, Maïté Garrouste-Orgeas, Alexandre Lautrette, Elie Azoulay, Bruno Mourvillier, Laurent Argaud, Laurent Papazian, Marc Gainnier, Dan Goldgran-Toledano, Samir Jamali, Anne-Sylvie Dumenil, Carole Schwebel, Jean-François Timsit, for the OUTCOMEREA study group, Jean-François Timsit, Elie Azoulay, Maïté Garrouste-Orgeas, Jean-Ralph Zahar, Christophe Adrie, Michael Darmon, Christophe Clec’h, Jean-Francois Timsit, Corinne Alberti, Stephane Ruckly, Sébastien Bailly, Lenka Styfalova, Aurélien Vannieuwenhuyze, Christophe Adrie, Bernard Allaouchiche, Laurent Argaud, Claire Ara-Somohano, Elie Azoulay, Francois Barbier, Julien Bohé, Christine Cheval, Christophe Clec’h, Michael Darmon, Anne-Sylvie Dumenil, Claire Dupuis, Jean-Marie Forel, Marc Gainier, Akim Haouache, Samir Jamali, Hatem Khallel, Alexandre Lautrette, Guillaume Marcotte, Eric Le Miere, Maxime Lugosi, Bruno Mourvillier, Benoît Misset, Delphine Moreau, Bruno Mourvillier, Laurent Papazian, Benjamin Planquette, Bertrand Souweine, Carole Schwebel, Nicolas Terzi, Gilles Troché, Marie Thuong, Guillaume Thierry, Dany Toledano, Eric Vantalon, Julien Fournier, Caroline Tournegros, Stéphanie Bagur, Mireille Adda, Vanessa Vindrieux, Loic Ferrand, Nadira Kaddour, Boris Berthe, Samir Bekkhouche, Kaouttar Mellouk, Sylvie Conrozier, Igor Theodose, Veronique Deiler, Sophie Letrou

**Affiliations:** 1 INSERM, U1042, Université Grenoble-Alpes, HP2, F-38000 Grenoble, France; 2 Service de Réanimation Médicale, Centre Hospitalier Universitaire Grenoble – Alpes, CS10217 Grenoble, cedex 09 France; 3 Medical Intensive Care Unit, Saint-Etienne University Hospital, Saint-Priest en Jarez, France; 4 Medical Intensive Care Unit, Nantes University Hospital Center, Nantes, France; 5 Department of Biostatistics, OUTCOMEREA™, Bobigny, France; 6 UMR 1137, Infection Antimicrobials Modelling Evolution (IAME) Team 5, Decision Sciences in Infectious Diseases (DeSCID), Control and Care, Sorbonne Paris Cité, Inserm/Paris Diderot University, Paris, France; 7 Polyvalent Intensive Care Unit, Groupe Hospitalier Saint-Joseph, Paris, France; 8 Medical Intensive Care Unit, Gabriel Montpied University Hospital, Clermont-Ferrand, France; 9 Service de Réanimation Médicale, CHU Saint-Louis, Assistance Publique – Hôpitaux de Paris, Paris, France; 10 Réanimation Médicale et Infectieuse, Hôpital Bichat Claude Bernard, Assistance Publique – Hôpitaux de Paris, Paris, France; 11 Medical Intensive Care Unit, Lyon University Hospital, Lyon, France; 12 Réanimation des Détresses Respiratoires et Infections Sévères, Hôpital Nord, Aix-Marseille University, Assistance Publique – Hôpitaux de Marseille, Unité de Recherche sur les Maladies Infectieuses et Tropicales Émergentes (URMITE), UMR CNRS 7278, Marseille, France; 13 Réanimation des Urgences et Medicale, CHU la Timone 2 Marseille, Aix-Marseille Université, 13385 Marseille, France; 14 Medical-Surgical Intensive Care Unit, Gonesse Hospital, Gonesse, France; 15 Medical-Surgical Intensive Care Medicine Unit, Dourdan Hospital, Dourdan, France; 16 Medical-Surgical Intensive Care Unit, Assistance Publique – Hôpitaux de Paris, Antoine Béclère University Hospital, Clamart, France; 17 Integrated Research Center, Inserm U1039, Radiopharmaceutical Bioclinical Mixed Research Unit, University Joseph Fourier, Grenoble, France

**Keywords:** Nutrition, Noninvasive mechanical ventilation, Intensive care unit, Acute respiratory failure, Pneumonia

## Abstract

**Background:**

Patients starting noninvasive ventilation (NIV) to treat acute respiratory failure are often unable to eat and therefore remain in the fasting state or receive nutritional support. Maintaining a good nutritional status has been reported to improve patient outcomes. In the present study, our primary objective was to describe the nutritional management of patients starting first-line NIV, and our secondary objectives were to assess potential associations between nutritional management and outcomes.

**Methods:**

Observational retrospective cohort study of a prospective database fed by 20 French intensive care units. Adult medical patients receiving NIV for more than 2 consecutive days were included and divided into four groups on the basis of nutritional support received during the first 2 days of NIV: no nutrition, enteral nutrition, parenteral nutrition only, and oral nutrition only.

**Results:**

Of the 16,594 patients admitted during the study period, 1075 met the inclusion criteria; of these, 622 (57.9%) received no nutrition, 28 (2.6%) received enteral nutrition, 74 (6.9%) received parenteral nutrition only, and 351 (32.7%) received oral nutrition only. After adjustment for confounders, enteral nutrition (vs. no nutrition) was associated with higher 28-day mortality (adjusted HR, 2.3; 95% CI, 1.2–4.4) and invasive mechanical ventilation needs (adjusted HR, 2.1; 95% CI, 1.1–4.2), as well as with fewer ventilator-free days by day 28 (adjusted relative risk, 0.7; 95% CI, 0.5–0.9).

**Conclusions:**

Nearly three-fifths of patients receiving NIV fasted for the first 2 days. Lack of feeding or underfeeding was not associated with mortality. The optimal route of nutrition for these patients needs to be investigated.

**Electronic supplementary material:**

The online version of this article (doi:10.1186/s13054-017-1867-y) contains supplementary material, which is available to authorized users.

## Background

Over the last two decades, noninvasive ventilation (NIV) has become the cornerstone supportive treatment for acute respiratory failure requiring intensive care unit (ICU) admission. NIV decreases the need for endotracheal mechanical ventilation (MV), which is a major source of complications. NIV has been proven to decrease mortality, particularly in patients experiencing acute exacerbations of chronic obstructive pulmonary disease (COPD) or acute cardiogenic pulmonary edema [1, 2]. Both acute respiratory failure and COPD may induce malnutrition, which may adversely affect patient outcomes [3–6]. However, no recommendations about nutritional support to patients receiving NIV are available.

Patients may be unable to remain off NIV for a sufficient time to allow adequate food ingestion or may feel too short of breath to eat. Either enteral nutrition via a nasogastric tube or parenteral nutrition can be used in this situation. Enteral nutrition has been associated with a lower infection rate than parenteral nutrition in patients with adequate gut function [7, 8]. However, enteral nutrition via a nasogastric tube is associated with adverse effects (e.g., nosocomial sinusitis and tracheobronchial aspiration of gastric contents) [9, 10], such as mask leakage with decreased NIV efficiency [11]. In a recent physiological study demonstrating improved breathing-swallowing coordination and reduced dyspnea with NIV during acute COPD exacerbations [12], an off switch added to the ventilator for use during swallowing proved effective. Such a device may allow patients to eat sufficiently, including during NIV sessions using a nasal mask, thus obviating the need for nasogastric tube feeding.

Despite the considerable emphasis placed recently on appropriate nutritional support in critically ill patients, the accurate evaluation of malnutrition and provision of adequate nutritional support remain major challenges in ICU patients [13, 14], especially those requiring NIV, for the above-mentioned reasons. The considerable body of evidence available on the nutritional management of patients receiving MV contrasts with the paucity of data on nutrition in patients started on NIV to treat acute respiratory failure.

To begin filling this knowledge gap, we performed a multicenter cohort study with the main objective of describing the nutritional management of patients admitted to the ICU and given first-line NIV. The secondary objectives were to assess potential associations between the type of nutritional management and patient outcomes, including the need for MV, occurrence of infection (i.e., bacteremia, urinary tract infection, ventilator-associated pneumonia [VAP], intensive care unit-acquired pneumonia [ICU-AP], central line-associated bloodstream infection), and death.

## Methods

We used the multicenter OUTCOMEREA™ database contributed to by physicians in 20 French ICUs with the assistance of trained database monitors, as described elsewhere [15]. This study was approved by our institutional review board (Clinical Investigation Center Ethics Committee [CECIC] Clermont-Ferrand IRB number 5891, reference number 2007-16), which waived the need for written informed consent of the participants, in accordance with French legislation on noninterventional studies. However, the patients and their next of kin were informed about the inclusion of their anonymized health data in the database, and none declined participation.

### Study population

We included adults admitted to medical ICUs between 2000 and 2015 who received NIV for more than 2 consecutive days. The 2-day period was selected as a criterion for providing nutritional support [16, 17]. Exclusion criteria were previous ICU admission during the same hospital stay, postoperative period, neuromuscular disease, NIV received after extubation, and treatment limitation decisions within 24 h after starting NIV. We separated the patients into four groups on the basis of nutrition during the first 2 days of NIV: no nutrition (NoN), enteral nutrition with or without parenteral nutrition (EN), parenteral nutrition only (PN), and oral nutrition only (ON).

### Outcomes

We assessed associations between nutritional groups and the need for MV, the occurrence of nosocomial infection (i.e., bacteremia, urinary tract infection, VAP, ICU-AP, central line-associated bloodstream infection), and death. A patient was considered as a candidate for invasive MV in case of loss of consciousness, hemodynamic instability, inability to maintain the airway, worsening respiratory distress, or persistently low peripheral arterial oxygen saturation (<90%) despite 100% fraction of inspired oxygen.

Nosocomial infection was defined as bacteremia, urinary tract infection, pneumonia, or catheter-related infection occurring after 72 h from admission. The definition of nosocomial infection was taken from the Hospital in Europe Link for Infection Control through Surveillance (HELICS) project [18]. Bacteremia was defined as the presence of pathogenic bacteria in blood culture. Catheter-related infection was defined as a positive quantitative catheter culture (≥ 10^3^ colony-forming units [CFU]/ml) treated by physicians in charge. ICU-AP was defined as a lower respiratory tract infection that was not incubating at the time of hospital admission, with symptom onset 2 or more days after NIV initiation. ICU-AP was suspected on the basis of new or progressive radiographic pulmonary infiltrates with at least two of the following: temperature > 38 °C or < 36 °C, leukocytosis > 10,000/mm^3^ or leukopenia < 4000/mm^3^, and purulent respiratory secretions [19]. The diagnosis was confirmed by a quantitative sputum culture > 10^5^ CFU/ml, bronchoalveolar lavage (BAL) culture > 10^4^ CFU/ml, or plugged telescoping catheter culture > 10^3^ CFU/ml. VAP was defined as persistent pulmonary infiltrates seen on chest radiographs combined with purulent tracheal secretions and/or body temperature ≥ 38.5 °C or ≤ 36.5 °C and/or peripheral blood leukocyte count ≥ 10,000/mm^3^ or ≤ 4000/mm^3^. A definite diagnosis of VAP required microbiological confirmation by quantitative culture from a protected specimen brush (> 10^3^ CFU/ml), plugged telescopic catheter specimen (> 10^3^ CFU/ml), BAL fluid specimen (> 10^4^ CFU/ml), or endotracheal aspirate (> 10^5^ CFU/ml). A urinary tract infection was defined as a quantitative culture containing ≥ 10^5^ CFU/ml of one or two organisms.

### Data collection

The baseline characteristics listed in Table 1 were collected at ICU admission. The McCabe and Jackson classification was used to estimate the prognosis of any preexisting disease (rapidly fatal, ultimately fatal, or nonfatal).

**Table 1:** Baseline characteristics of the 1075 patients

	No. (%) or median [IQR]				
Baseline characteristics	No nutrition (*n* = 622)	Parenteral nutrition (*n* = 74)	Enteral nutrition (*n* = 28)	Oral nutrition (*n* = 351)	*P* value*
Age, years (1 missing)	70.4 [59.4–80.2]	67.3 [56.4–78.8]	66.6 [60.9–77.3]	71.6 [59.4–80.3]	0.39
Male sex	384 (61.7)	47 (63.5)	19 (67.9)	206 (58.7)	0.64
Hospital LOS before ICU admission, days	1 [1–3]	1.5 [1–5]	2 [1–6.5]	1 [1–2]	0.08
Weight, kg (162 missing)	73 [61–87]	71 [62–88]	61 [55–74]	73 [60–85]	0.06
BMI, kg/m^2^ (270 missing)	26 [22.8–30.9]	25 [22.2–30.2]	23.4 [19.2–26.7]	25.5 [21.8–30.5]	0.09
Admission diagnosis (12 missing)					**<0.01**
COPD exacerbation	98 (16)	8 (11.1)	2 (7.4)	93 (26.6)	
Acute respiratory failure	366 (59.6)	46 (63.9)	13 (48.1)	183 (52.3)	–
Coma as referral diagnosis related to hypercapnia	21 (3.4)	2 (2.8)	5 (18.5)	7 (2)	–
Miscellaneous	129 (21)	16 (22.2)	7 (25.9)	67 (19.1)	
Chronic disease^a^					
Heart failure	144 (23.2)	15 (20.3)	4 (14.3)	87 (24.8)	0.55
Respiratory failure	256 (41.2)	32 (43.2)	6 (21.4)	186 (53)	**<0.01**
Hepatic failure	25 (4)	3 (4.1)	0 (0)	8 (2.3)	0.36
Renal failure	42 (6.8)	4 (5.4)	3 (10.7)	19 (5.4)	0.64
Immunosuppression	112 (18)	13 (17.6)	4 (14.3)	39 (11.1)	**0.04**
McCabe and Jackson classification (14 missing)					
Nonfatal	277 (45.3)	35 (48.6)	11 (40.7)	159 (45.4)	0.38
Ultimately fatal disease	287 (46.9)	31 (43.1)	13 (48.1)	176 (50.3)	
Fatal within 1 year	48 (7.8)	6 (8.3)	3 (11.1)	15 (4.3)	
Charlson comorbidity index	2 [1–3]	1 [1–3]	1.5 [0.5–3]	2 [1–3]	0.22
SAPS II	37 [30–47]	35.5 [26–45]	43.5 [34.5–50.5]	33 [25–42]	<0.01
SOFA	4 [2–5]	4 [3–6]	4.5 [3.5–7]	4 [2–5]	**0.02**

*Abbreviations: LOS* length of stay, *ICU* intensive care unit, *BMI* Body Mass Index, *COPD* chronic obstructive pulmonary disease, *SAPS II* Simplified Acute Physiology Score II, *SOFA* Sequential Organ Failure Assessment

^a^Assessed using the Knaus scale

*Comparaison between the groups

### Quality of the database

For most of the study variables, the data capture software immediately ran an automatic check for internal consistency, generating queries that were sent to the ICUs for resolution before incorporation of the new data into the database. In each participating ICU, data quality was checked by having a senior physician from another participating ICU review a 2% random sample of the study data from alternate years. A 1-day-long data capture training course held once annually was open to all OUTCOMEREA^TM^ investigators and study monitors.

### Statistical analysis

Characteristics of patients and outcomes were described using frequency and percentage for qualitative variables and median and interquartile range for quantitative variables. A multivariable Cox model was built to evaluate potential associations between nutrition group (NoN, EN, PN, or ON) and day 28 mortality. To assess potential associations linking nutrition group to the use of MV and to the occurrence of nosocomial infection (i.e., bacteremia, urinary tract infection, VAP, ICU-AP, central line-associated bloodstream infection) within 28 days, we used a multivariable Fine and Gray model to allow for discharge alive from the ICU as a competing event. The adequacy of the models was checked using the graphical and numerical methods of Lin et al. (1993). We looked for associations between nutrition group and ventilator-free days by day 28 using multivariable negative binomial regression. Finally, an association between nutrition group and the change in partial pressure of carbon dioxide (PaCO_2_) from ICU admission to the end of the second day (ΔPaCO_2_) was sought using analysis of variance in the 772 patients with available ΔPaCO_2_ data.

All models were adjusted on clinically relevant variables. Choices among collinear variables were based on clinical relevance, rate of missing variables, and reproducibility of definitions. The variables selected for adjustment were age, sex, Sequential Organ Failure Assessment (SOFA) score on the first day of NIV, chronic diseases, diabetes, obesity, main diagnosis at ICU admission, respiratory and neurologic SOFA subscores at ICU admission, and hospital length of stay before ICU admission shorter than 2 days. Missing values occurred for age (*n* = 1) and main diagnosis at ICU admission (*n* = 19) and were imputed using the median and mode, respectively.

*P* values less than 0.05 were considered significant. Statistical analyses were performed using SAS version 9.4 software (SAS Institute, Cary, NC, USA).

## Results

### Patients

**Fig. 1:**
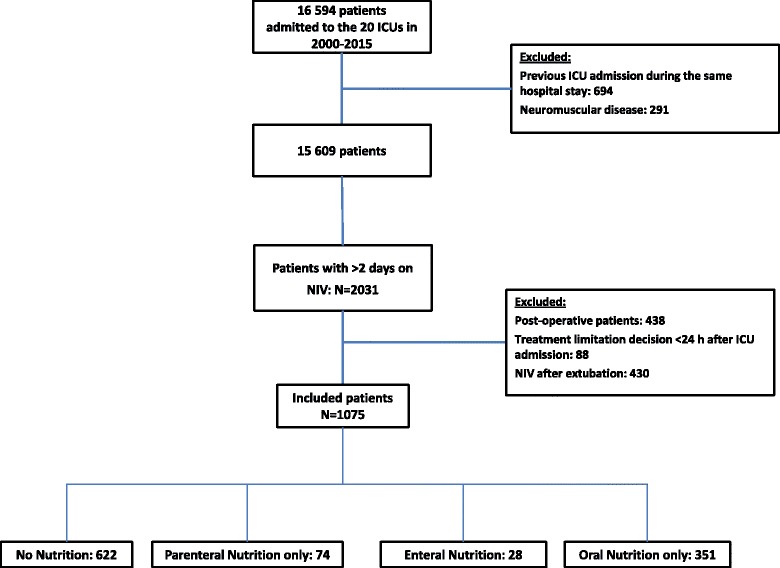
Flowchart of the study. *ICU* Intensive care unit, *NIV* Noninvasive ventilation

During the study period, of the 16,594 patients recorded in the database, 1075 (6.5%) required first-line NIV for longer than 2 days. As shown in Table 1, NIV was performed for COPD exacerbation in 201 patients (19%), acute respiratory failure in 608 patients (57%), and hypoventilation associated with coma in 35 patients (3%). During the first 2 days of NIV, 622 patients (57.8%) received no nutrition, 28 (2.6%) received EN, 74 (6.9%) received PN only, and 351 (32.7%) received ON only (Fig. 1).

Table 1 reports the main patient characteristics. Nutrition groups differed significantly regarding ICU admission category, main symptom at ICU admission, body weight, severity, and length of hospital stay before ICU admission. Acute illness severity as assessed by the Simplified Acute Physiology Score II was lower in the ON group than in the NoN group. The median ICU length of stay was 4 days (interquartile range, 2–7).

### Associations with type of nutrition

Overall, within the first 28 days, 145 patients (13.5%) developed nosocomial infection (Additional file 1: Table S1), 158 (14.7%) required MV, and 161 (15.0%) died. Eighty-nine patients (8.3%) developed ICU-AP, and 42 patients (3.9%) developed VAP. Table 2 reports the results of the multivariable analyses.

**Table 2:** Adjusted analysis of associations between type of nutrition and four 28-day outcomes: invasive mechanical ventilation, mortality, ventilator-free days, and intensive care unit-acquired pneumonia

28-Day outcomes	sHR (95% CI)	*P* value
Invasive mechanical ventilation		**<0.0001**
No nutrition	1.0	
Parenteral nutrition	**1.7 (1.0–2.7)**	**0.04**
Enteral nutrition	**2.1 (1.1–4.2)**	**0.03**
Oral nutrition	0.5 (0.3–0.7)	**0.0005**
	HR (95% CI)	*P* value
Mortality		**0.02**
No nutrition	1.0	
Parenteral nutrition	1.3 (0. 7–2.2)	0.39
Enteral nutrition	**2.3 (1.2–4.4)**	**0.01**
Oral nutrition	0.8 (0.5–1.1)	0.20
	RR (95% CI)	*P* value
Ventilator-free days		**0.006**
No nutrition	1.0	
Parenteral nutrition	**0.9 (0.7–1.0)**	**0.11**
Enteral nutrition	**0.7 (0.5–0.9)**	**0.01**
Oral nutrition	1.1 (1.0–1.2)	0.10
	sHR (95% CI)	*P* value
Nosocomial infection^a^		**0.02**
No nutrition	1.0	
Parenteral nutrition	1.0 (0.5–1.9)	0.96
Enteral nutrition	**2.2 (1.1–4.5)**	**0.03**
Oral nutrition	0.7 (0.5–1.0)	0.06
	sHR (95% CI)	*P* value
ICU-acquired pneumonia		0.18
No nutrition	1.0	
Parenteral nutrition	1.1 (0.5–2.5)	0.75
Enteral nutrition	2.1 (0.8–5.4)	0.13
Oral nutrition	0.7 (0.4–1.2)	0.17
	sHR (95% CI)	*P* value
VAP		**0.002**
No nutrition	1.0	
Parenteral nutrition	**2.9 (1.1–7.4)**	**0.03**
Enteral nutrition	**6.9 (2.1– 22.3)**	**0.001**
Oral nutrition	0.9 (0.4–2.1)	0.76

*Abbreviations: sHR* Subdistribution hazard ratio, *ICU* Intensive care unit, *RR* Relative risk, *VAP* Ventilator-associated pneumonia

Results are given as HR for Cox models, RR for the negative binomial model, and subdistribution hazard ratio (sHR) for the Gray and Fine model. (i.e., age, sex, hospital length of stay before ICU admission < 2 days, acute illness severity at ICU admission [Sequential Organ Failure Assessment {SOFA} score], respiratory and neurologic SOFA subscores at ICU admission, obesity, chronic disease, and main diagnosis at ICU admission)

^a^Nosocomial infection includes bacteremia, urinary tract infection, VAP, ICU-acquired pneumonia, central line-associated bloodstream infection

**Fig. 2:**
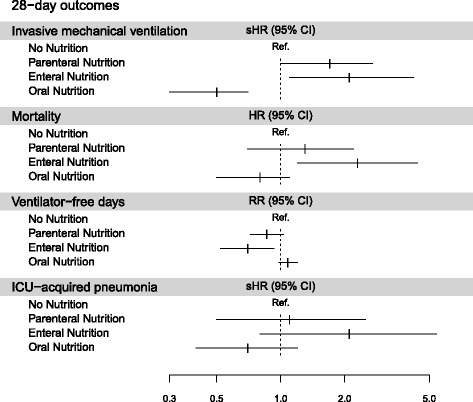
Impact of nutrition group on outcome. *ICU* Intensive care unit, *sHR* Subdistribution hazard ratio, *RR* Relative risk

After adjustment for confounders (i.e., age, sex, hospital stay length before ICU admission < 2 days, acute illness severity at ICU admission [SOFA score], respiratory and neurologic SOFA subscores at ICU admission, obesity, chronic disease, and main diagnosis at ICU admission), nutrition group was independently associated with higher incidence of nosocomial infection (*P* = 0.02), VAP (*P* = 0.002), and 28-day mortality (*P* = 0.02) (Fig. 2). The incidence of infection was higher in the EN group than in the NoN group (HR, 2.2; 95% CI, 1.1–4.5; *P* = 0.03). The occurrence of nosocomial infection was not different in the PN or ON group compared with the NoN group. The nutrition group was not associated with the occurrence of ICU-AP (*P* = 0.18). The incidence of VAP was higher in the EN and PN groups than in the NoN group (HR, 6.9; 95% CI, 2.1–22.3; *P* = 0.001; and 2.9; 95% 95% CI, 1.1–7.4; *P* = 0.03, respectively).

Mortality was higher in the EN group than in the NoN group (HR, 2.3; 95% CI, 1.2–4.4; *P* = 0.01). Mortality on day 28 was not different in the PN or ON group compared with the NoN group. The number of ventilator-free days by day 28 was significantly associated with nutrition group (*P* = 0.006). Compared with NoN, EN was associated with fewer ventilator-free days by day 28 (RR per day, 0.7; 95% CI, 0.5–0.9). ΔPaCO_2_ over the first 2 days was not associated with nutrition group.

## Discussion

In this large, multicenter database study, nearly three-fifths of patients fasted during the first 2 days of first-line NIV used to treat acute respiratory failure in the ICU. About one-third of patients received oral nutrition. EN and PN were rarely used but were more often associated with the need for invasive MV. EN, but not fasting, was associated with higher 28-day mortality. EN was also associated with a more days on ventilation. Type of nutrition was not associated with the occurrence of ICU-AP.

In a previous observational study, patients with acute respiratory failure requiring NIV often had inadequate oral food intake, particularly early during admission and as NIV duration increased [20]. Although nutritional support is now recognized as an essential component of the management of critically ill patients [17, 21, 22], the optimal intake and timing remain unclear. Positive associations between protein intake and survival have been reported in observational studies [23, 24]. A major weakness of these studies is the heterogeneity in acute illness severity, which is a key potential confounder. Patients with lower severity tolerate EN better, have caloric and protein intake closer to the optimal values, and experience better outcomes. Moreover, recent methodologically sound and adequately powered randomized controlled trials have not generated unequivocal evidence that full-replacement nutrition early in the course of critical illness produces clinical benefits [25, 26].

In addition to timing of initiation, the delivery route is viewed as an important determinant of the effect of nutritional support. EN may produce nonnutritional benefits, including maintenance of structural and functional gut integrity and of the gut microbiome [27, 28]. However, EN may fail to provide sufficient nutrition, particularly at the acute phase of critical illness and in patients with gastrointestinal dysfunction [29, 30]. PN may be more effective in achieving nutritional targets but is associated with infections, which are probably related to overnutrition and hyperglycemia, as shown in two meta-analyses [31, 32]. These clinical data have translated into a consensus, expressed in both international recommendations [17, 21] and expert opinions [33, 34], that EN should be preferred in critically ill patients without contraindications to this route. CALORIE is a large, randomized trial in which researchers recently compared EN and PN started within 36 h after ICU admission and continued for up to 5 days in unselected ICU patients [35]. In this pragmatic trial involving 2388 patients, neither 30-day mortality nor the frequency of infections differed between the two groups. These results challenge the previously held belief that EN produces better outcomes than PN in ICU patients.

Enteral nutrition via nasogastric tube and parenteral nutrition was rarely used in our study. As previously described, enteral nutrition via a nasogastric tube is associated with adverse effects [9, 10], such as mask leakage with decreased NIV efficiency [11]. Furthermore, Kogo et al. observed in a retrospective study that enteral nutrition caused more airway complications in subjects receiving NIV for acute respiratory failure than in those receiving nutrition by other routes [36]. We can hypothesize that physicians promote NIV efficiency rather than nutrition during the first 2 days of NIV. These results are in accordance with those of previous studies. Indeed, Bendavid et al. showed, in a large prevalence study of nutrition practice in intensive care, that enteral feeding was prescribed to only 10% of the patients on the first day, but this number increased to more than 40% of the patients after 5 days [37].

Parenteral nutrition was used for only 7% of the patients. As suggested earlier, some physicians could be reluctant to use enteral nutrition via a nasogastric tube for patients with NIV. Moreover, a fluid-restricted strategy targeting patients with acute respiratory failure (more than half of included patients) may explain few parenteral nutrition prescriptions in the study population [38]. Here, type of nutrition was not associated with the occurrence of ICU-AP. However, occurrence of VAP was higher in the PN and EN groups. This result is coherent with the fact that the EN and PN groups were associated with a higher need for invasive MV. Indeed, a protective effect of NIV has been reported in single-center [39] and multicenter [40] studies and can be attributed both to the absence of an endotracheal tube bypassing the upper airways and to decreased invasiveness of the overall management. Contrary to previous results, the reported increase in infectious complications that have been associated with the parenteral route was not observed [41]. The absence of significance of this result is probably due to the lack of power, and other contributory reasons may be improvements in current management of vascular access [42].

In our study, fasting for 48 h after NIV initiation was not associated with 28-day mortality. In contrast, EN and PN during the first 2 days on NIV were associated with higher need for invasive MV, and EN was associated with higher 28-day mortality. The observational design of this study could not prove causality. Critically ill patients may be unable to tolerate EN, which may be associated with a higher risk of respiratory complications (e.g., mucus plug, aspiration pneumonia) [36]. EN may increase the residual gastric volume, thereby promoting bacterial colonization and the risk of aspiration pneumonia, and VAP may increase [43, 44]. Many risk factors for aspiration pneumonia have been identified, including intubation and difficult intubation, peri-intubation vomiting, anesthesia, older age, gastroparesis, reflux, impaired swallowing, mental state alteration, and cardiac arrest [45]. In our study, type of nutrition was not associated with the occurrence of ICU-AP. However, occurrence of VAP was higher in the PN and EN groups. This result is coherent with the fact that the EN and PN groups were associated with greater need for invasive MV. Moreover, nosocomial infection was higher in the EN group. We can hypothesize that the higher mortality was due to higher occurrence of nosocomial infection. We cannot exclude the negative impact of overfeeding. As previously demonstrated, high protein intake may lead to azotemia, hypertonic dehydration, and metabolic acidosis [46]. Large amounts of intravenous glucose may result in hyperglycemia, hypertriglyceridemia, and hepatic steatosis [47], which can, however, be prevented to a considerable extent by insulin therapy targeting normoglycemia [48]. Moreover, NIV decreases the work and therefore the energy needs of the diaphragm [49]. Previous data suggest that oversupply of energy relative to needs may worsen diaphragmatic dysfunction [50].

Our study has several limitations. The patients were identified retrospectively from the database, and we cannot exclude selection bias. Choice of nutrition route was made by clinicians without a specific protocol. Moreover, unfortunately, we cannot provide the amount of calories and protein for each group. NIV practices may have varied across the study centers. The indications to perform NIV were heterogeneous but in accordance with recommendations. Decisions of application (i.e., continuous session, intermittent) were made by attending clinicians, and total duration of NIV session was not assessed; thus, we cannot exclude the possibility that these modalities influenced the choice of the route of nutrition. However, our study was a multicenter study in 20 French ICUs, and our results are in accordance with previous results. Reeves et al. demonstrated that patients with acute respiratory failure requiring NIV often had inadequate oral intake, particularly with increasing time on NIV and earlier during their hospital admission [20]. Moreover, a retrospective study can detect associations but cannot provide evidence of causality. Although we adjusted for potential confounders, we cannot exclude the possibility that the observed associations were related to a clustering effect (i.e., to variables that were not adjusted for and that were related both to the choice of nutrition type and to outcomes).

## Conclusions

Most patients received no nutrition at all during the first 2 days of first-line NIV. Early EN in a heterogeneous population was independently associated with higher 28-day mortality and fewer ventilator-free days. Additional studies are required to assess the need for, as well as the best timing and route of, nutritional support in this specific population.

## Additional file

Additional file 1: Table S1. Description of interface used for NIV and type of exhibited complications according to nutritional groups. (DOCX 17 kb)

## Abbreviations

BAL: Bronchoalveolar lavage
BMI: Body mass index
CFU: Colony-forming units
COPD: Chronic obstructive pulmonary disease
EN: Enteral nutrition
ICU: Intensive care unit
ICU-AP: Intensive care unit-acquired pneumonia
LOS: Length of stay
MV: Mechanical ventilation
NIV: Noninvasive ventilation
NoN: No nutrition
ON: Oral nutrition
PaCO_2_: Partial pressure of carbon dioxide
PN: Parenteral nutrition
RR: Relative risk
SAPS II: Simplified Acute Physiology Score II
sHR: Subdistribution hazard ratio
SOFA: Sequential Organ Failure Assessment
VAP: Ventilator-associated pneumonia
