# Supplementary material for: Initial nutritional management during noninvasive ventilation and outcomes: a retrospective cohort study
Source: Crit Care. 2017 Nov 29;21:293. doi: 10.1186/s13054-017-1867-y (PMC5707783; doi:10.1186/s13054-017-1867-y)
Supplement: Additional file 1: Table S1. — Description of interface used for NIV and type of exhibited complications according to nutritional groups. (DOCX 17 kb) [file 13054_2017_1867_MOESM1_ESM.docx]

| N (%) or median [IQR] | | | | | | | |
| --- | --- | --- | --- | --- | --- | --- | --- |
| **Variable (n miss)** | **No nutrition**  n=622 | **Parenteral nutrition**  n=74 | **Enteral nutrition**  n=28 | **Oral nutrition**  n=351 | ***P* value*** |  |  |
| Interface used for NIV |  |  |  |  | 0.22 |  |  |
| Not Specified | 191 (30.7) | 19 (25.7) | 9 (32.1) | 97 (27.6) |  |  |  |
| Facial Mask | 400 (64.3) | 55 (74.3) | 16 (57.1) | 235 (67) |  |  |  |
| Nasal Mask | 31 (5) | 0 (0) | 3 (10.7) | 19 (5.4) |  |  |  |
| ICU-AP | 53 (8.5) | 9 (12.2) | 5 (17.9) | 22 (6.3) | 0.08 | |  |
| VAP | 21 (3.4) | 8 (10.8) | 4 (14.3) | 9 (2.6) | **<.01** | |  |
| Bacteremia | 23 (3.7) | 6 (8.1) | 1 (3.6) | 9 (2.6) | 0.15 | |  |
| Urinary tract Infection | 33 (5.3) | 5 (6.8) | 3 (10.7) | 13 (3.7) | 0.29 | |  |
| Central-line associated bloodstream infection | 15 (2.4) | 3 (4.1) | 1 (3.6) | 4 (1.1) | 0.33 | |  |
| Patients requiring mechanical ventilation | 100 (16.1) | 21 (28.4) | 10 (35.7) | 27 (7.7) | **<.01** | |  |

Table S1 : Description of interface used for NIV and type of exhibited infections according to nutritional groups

Abbreviations: VAP: ventilator associated infection; ICU: ICU- acquired pneumonia; ICU: intensive care unit; NIV: non invasive ventilation
